# Digitally Assessed Long COVID Symptomatology Is Associated With Lymphocyte Mitochondrial Dysfunction and Altered Immune Potential

**DOI:** 10.1093/ofid/ofaf447

**Published:** 2025-11-17

**Authors:** Vasile Mihai Sularea, Liam Townsend, Cian Reid, Andreea V Atanasescu, Adam H Dyer, Federica Giangrazi, Roman Rocha Lawrence, Manoj Sivan, Barry Moran, Derek G Doherty, Niall P Conlon, Aideen Long, Cliona O’Farrelly

**Affiliations:** School of Biochemistry and Immunology, Trinity Biomedical Sciences Institute, Trinity College Dublin, Dublin, Ireland; Department of Infectious Diseases, St James’s Hospital, Dublin, Ireland; Department of Clinical Medicine, School of Medicine, Trinity Translational Medicine Institute, Trinity College Dublin, Dublin, Ireland; School of Biochemistry and Immunology, Trinity Biomedical Sciences Institute, Trinity College Dublin, Dublin, Ireland; School of Biochemistry and Immunology, Trinity Biomedical Sciences Institute, Trinity College Dublin, Dublin, Ireland; Discipline of Medical Gerontology, School of Medicine, Trinity College Dublin, Dublin, Ireland; Trinity Translational Medicine Institute, Trinity College Dublin, Dublin, Ireland; School of Biochemistry and Immunology, Trinity Biomedical Sciences Institute, Trinity College Dublin, Dublin, Ireland; ELAROS 24/7 Limited, Sheffield S1 2BJ, UK; Leeds Institute of Rheumatic and Musculoskeletal Medicine, School of Medicine, University of Leeds, Leeds, UK; School of Biochemistry and Immunology, Trinity Biomedical Sciences Institute, Trinity College Dublin, Dublin, Ireland; Trinity Translational Medicine Institute, Trinity College Dublin, Dublin, Ireland; Department of Immunology, School of Medicine, Trinity College Dublin, Dublin, Ireland; Department of Immunology, School of Medicine, Trinity College Dublin, Dublin, Ireland; Department of Immunology, St James’s Hospital, Dublin, Ireland; Wellcome-HRB Clinical Research Facility, St James’s Hospital, Dublin, Ireland; Trinity Translational Medicine Institute, Trinity College Dublin, Dublin, Ireland; School of Biochemistry and Immunology, Trinity Biomedical Sciences Institute, Trinity College Dublin, Dublin, Ireland; School of Medicine, Trinity College Dublin, Dublin, Ireland

**Keywords:** Immunometabolism, Long COVID, Lymphocyte, Mitochondria, Natural Killer cell

## Abstract

**Background:**

Postacute sequelae of SARS-CoV-2 infection, also known as long COVID (LC), is a complex and heterogenous condition affecting millions worldwide with a poorly understood underlying pathology. Although metabolic dysregulations have been described in LC, it remains unclear whether circulating immune cells exhibit immunometabolic alterations.

**Methods:**

We conducted a detailed clinical, immunologic, and mitochondrial analysis on 27 patients with LC and 27 who recovered from COVID-19 and were healthy. Symptom burden and severity were assessed and quantified via a digital platform with the modified COVID-19 Yorkshire Rehabilitation Scale. Mitochondrial function of circulating immune cell populations (lymphocytes and monocytes) was analyzed by measuring mitochondrial mass and mitochondrial membrane potential. Production of 11 cytokines after whole blood stimulation with bacterial and viral agonists was measured by multiplex immunoassay. Relationships between mitochondrial and immune parameters with LC symptoms were investigated.

**Results:**

Patients with LC exhibited significant symptom burden, with worsening across all symptom domains as compared with their health state before SARS-CoV-2 infection. They also had a decreased mitochondrial membrane potential of CD56^bright^ natural killer cells, particularly in patients experiencing dizziness, whereas reduced mitochondrial membrane potential in CD4+ lymphocytes was found in patients with worsening breathlessness. Upon LPS stimulation, patients with LC demonstrated significantly lower IFN-γ production. In response to viral ligand R848, impaired IFN-β and IL-10 responses were associated with worsening cough and executive functions.

**Conclusions:**

Symptom severity in LC is associated with immune cell mitochondrial dysfunction and altered cytokine responses, highlighting potential disease biomarkers and targets for future therapeutic strategies.

The COVID-19 pandemic, caused by the SARS-CoV-2 virus, is associated with significant morbidity and mortality during acute infection. As a result of developing therapeutics, as well as improved understanding of the underlying immunology driving acute COVID-19 and increased immunity from vaccination, acute mortality and morbidity rates have dramatically declined across the globe [1–3]. However, ongoing ill-health following resolution of acute infection, commonly referred to as long COVID (LC), persists in 3% to 10% with as many as 400 million people globally estimated to be currently living with LC [4, 5]. The features of LC are heterogenous, with fatigue, postexertional malaise, cognitive dysfunction, and dysautonomia being the most commonly reported symptoms [6, 7]. Despite the burden of disease and its associated functional impairment, the pathology underlying LC remains poorly understood [8]. As a result, therapeutic targets are lacking, and current approaches revolve around symptom management and improving function via rehabilitation approaches [9].

Multiple mechanisms for the underlying pathology of LC have been proposed, including endothelial dysfunction, autoimmunity, and persistent immune activation [10–12]. However, studies to date have shown conflicting and contradictory evidence, with elements of immune dysregulation, immune activation, and immunometabolism demonstrated but no consistent observations [13, 14]. In some cases, observed immune changes were distinct from ongoing symptoms [15, 16]. These differences may in part be due to LC being an umbrella term for the end points of multiple pathologic pathways.

In addition to the immunologic abnormalities often associated with LC, metabolic abnormalities have been proposed as being responsible for many of the symptoms seen [17]. Dysfunctional mitochondria are particularly amenable to study in immune cell populations, where they are associated with immune cell dysfunction [18]. Mitochondrial function in immune cells can be assessed via flow cytometry through measurement of mitochondrial mass and mitochondrial membrane potential (MMP) [19, 20]. Measuring mitochondrial mass and MMP is critical for distinguishing among different types of mitochondrial dysfunction. While an increase in mitochondrial mass may suggest mitochondrial biogenesis or accumulation of damaged mitochondria, membrane potential reflects the ability of mitochondria to sustain cellular energy demands. The absence of correlation between these parameters suggests a potential decoupling of mitochondrial mass and functional capacity. This discrepancy may indicate that an increase in mitochondrial content does not necessarily translate to improved mitochondrial function, possibly due to defective oxidative phosphorylation or accumulation of dysfunctional mitochondria. Given the crucial role of mitochondria in immune cell function [21], understanding this relationship may provide insights into immune dysregulation in LC and its impact on symptom severity and distinct clinical phenotypes.

The heterogeneity of symptoms associated with LC presents an ongoing challenge to these studies, and comprehensive characterization of symptoms is necessary for any subsequent immunologic investigations. Here, we use the modified COVID-19 Yorkshire Rehabilitation Scale (C19-YRSm), a patient-recorded outcome measure (PROM) developed to capture and quantify persistent symptoms of LC with questions spanning all aspects of the 2001 WHO International Classification of Functioning, Disability and Health Framework [22, 23]. The C19-YRSm is hosted on a cloud-based digital platform developed by a third-party digital health company (ELAROS 24/7 Ltd) in collaboration with researchers at the University of Leeds [24]. The platform enables remote assessment, triage, monitoring, and management of patients via a clinical web portal and scalable patient-facing application with a suite of configurable features, including the C19-YRSm. The C19-YRSm is clinically validated and recognized internationally, and it has been adopted globally, forming part of national strategies and guidelines [25]. We subsequently investigated if dysfunctional mitochondria within circulating immune cells are contributors to LC and, furthermore, whether these dysfunctional mitochondria and associated changes in immune responses are related to LC symptom severity and distinct clinical phenotypes.

## METHODS

### Study Setting and Participants

Participants with LC attending the post–COVID-19 clinic at St James's Hospital, Dublin, Ireland, were enrolled in the STTAR Bioresource (St James's Hospital, Tallaght University Hospital, and Trinity College Dublin Allied Researchers) [26]. This clinic sees patients with ongoing symptoms lasting >6 months following COVID-19 infection. Participants were invited to take part during a 4-month period from May to August 2024. Eligible participants required (1) a positive result from either a self-taken antigen test or a laboratory-performed polymerase chain reaction test for COVID-19 and (2) the index COVID-19 infection to have occurred at a minimum 1 year prior to recruitment. Participants were excluded if they were taking any immunomodulatory medication or had evidence of an active infection. Time since infection, comorbidities, and comedications were recorded at the time of recruitment, as well as demographic data including sex, age, and ethnicity. Severity of initial infection was scored by the World Health Organization ordinal scale [27]. A COVID-19–recovered healthy (CRH) cohort was also recruited. This cohort consisted of staff members within our institutions who had confirmed COVID-19 infection, either by polymerase chain reaction or self-taken antigen test, and reported full recovery with no symptoms consistent with LC.

### Clinical Assessment

PROMs were recorded with ELAROS, a third-party digital platform [24]. The C19-YRSm was completed by all participants. It comprises 17 items across multiple symptom and functional ability domains scored on a 4-point response category (0–3): 0, symptom not present; 1, a mild problem (not affecting daily life); 2, a moderate problem (affecting daily life to a certain extent); and 3, a severe problem (life disturbing or affecting all aspects of daily life). Participants were asked to describe their symptom and functional state preinfection and now. The symptoms include those classically associated with LC (eg, fatigue, breathlessness, postexertional malaise, and neurocognitive issues) as well as broader functional questions regarding communication, personal care, and socializing. The C19-YRSm generates a score for each symptom as well as a symptom severity subscale (questions 1–10, score 0–30), a functional disability subscale (questions 11–15, score 0–15), and an additional symptom burden subscale (0–25). Finally, an overall health score (0–10) is calculated, with lower scores indicative of poorer overall health.

### Blood Processing

Whole blood was collected in lithium-heparin–coated tubes (BD Vacutainer 367526). Blood samples were processed the same day of collection: 5 mL of blood was used for the whole blood stimulation assays, while the remaining blood was used for peripheral blood mononuclear cell (PBMC) isolation and further immunophenotyping. For PBMC isolation, whole blood was diluted in phosphate-buffered saline (PBS) at a 1:1 ratio and gently layered on top of 10 mL of Ficoll-Paque Plus (Cytiva 17144993). The tubes were subsequently centrifuged for 15 minutes at 800*g* with deceleration set to 1. The PBMC layer was isolated and washed in PBS, and cells were counted for subsequent staining.

### Whole Blood Stimulation With Immunocheck Assay

Whole blood stimulation was performed as previously described [28]. Briefly, 1 mL of whole blood was diluted 3-fold in a S-Monovette tube (Sarsedt Ltd) prefilled with 2 mL of RPMI supplemented with 50-µg/mL streptomycin and 2.5-µg/mL amphotericin B (Thermo Fisher Scientific). Prefilled tubes contained medium only (untreated), lipopolysaccharide (LPS) as a Toll-like receptor 4 (TLR4) agonist (20 ng/mL), poly I:C as a TLR3 agonist (20 μg/mL), R848 as a TLR7/TLR8 agonist (1 μm), or IFN-α (1000 IU/mL). Samples were subsequently placed in an incubator at 37 °C for 22 hours. Following incubation, supernatants were collected by centrifugation of tubes at 600*g* for 10 minutes. Supernatants were stored at −80 °C for further analysis. Cytokine levels were measured with Luminex technology (MAGPIX System) according to the manufacturer’s instructions (Bio-Techne; R&D Systems).

### Mitochondrial and Extracellular Staining

Freshly isolated PBMCs were plated at 1–2 × 10^6^ cells per tube. Counting beads (10 μL/tube; Invitrogen C36950) were added, and cells were washed once in RPMI. Cells were resuspended in RPMI containing MitoTracker Green (final concentration, 100 nM; Invitrogen) to assess mitochondrial mass and tetramethylrhodamine methyl ester (final concentration 100 nM; Invitrogen) to measure MMP. Cells were incubated for 20 minutes at 37 °C for mitochondrial staining. Cells were further washed once with PBS and resuspended in near-infrared live/dead staining (1:500 in PBS; BioLegend) for live-dead staining (15 minutes at room temperature). Cells were further washed in FACS buffer (5% fetal bovine serum in PBS) and resuspended in the cocktail of extracellular staining antibodies specific for CD3, CD4, CD8, CD14, CD16, CD19, CD56, and HLA-DR (in FACS buffer; 5% fetal bovine serum in PBS) for extracellular staining (30 minutes at room temperature). Subsequently, cells were washed once in FACS buffer and resuspended in a final volume of 200 μL of FACS buffer. Cells were analyzed on the Aurora BD flow cytometer, and data were analyzed by FlowJo software (BD Biosciences). Gating strategy and antibody information are provided in Supplementary Figure 1 and Supplementary Table 1, respectively.

### Patient Consent Statement

Informed written consent was obtained from all participants in the current study in accordance with the Declaration of Helsinki. Ethical approval was obtained from the Tallaght University Hospital/St James Hospital Joint Research Ethics Committee (reference 2020-04 [01]).

### Statistical Analysis

Descriptive statistics are reported as means (SD) and median (IQR) as appropriate. Between-group differences were assessed with *t* tests, χ^2^ tests, and Wilcoxon rank sum tests as appropriate. Pre- and postinfection C19-YRSm scores were compared by Wilcoxon signed rank tests. Associations between symptom severity and mitochondrial function and between symptom severity and cytokine levels were analyzed via a generalized linear model, adjusting for age and sex, with subsequent correction for multiple testing (Bonferroni). All statistical analysis was carried out in R studio or Prism version 10 (GraphPad), and statistical significance was considered at *P* < .05 or an adjusted *P* value (*q*) < .1. Figures were produced with Prism version 10 and ggplot2 with R studio.

## RESULTS

### Participant Characteristics and Symptom Severity

**Figure 1.**
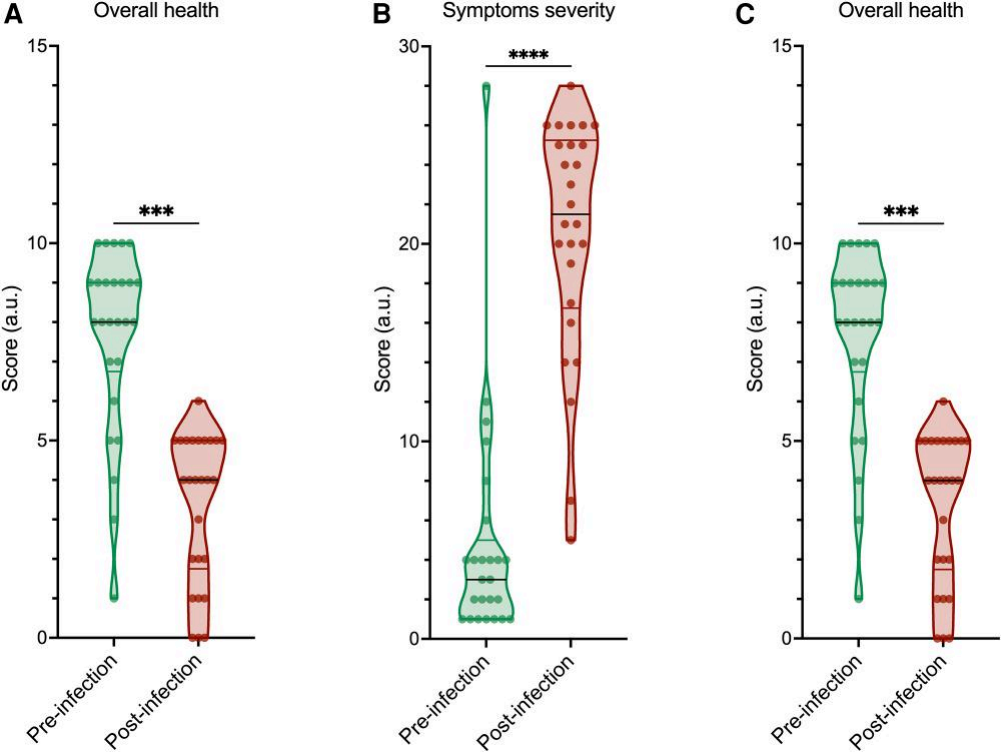
Changes in health before and after COVID-19 in patients with long COVID, including symptom burden and functional ability. *A–C*, Symptom severity, functional ability, and overall health scores before and after SARS-CoV-2 infection, as reported by the modified COVID-19 Yorkshire Rehabilitation Scale. Central black line, median; top and bottom lines, 75th and 25th percentiles. *P* values were calculated by 2-tailed paired *t* test. ****P < .0001. ***P < .01.

A cohort of 27 patients with LC were recruited from the LC Clinic at St James's Hospital. The majority were female (n = 19, 70%) and the median age was 52 years (IQR, 41–61.5). A matched CRH cohort of 27 individuals was also recruited. No participants received corticosteroid or any other immunomodulatory treatment during their acute illness. The median duration of LC symptoms was 1118 days (IQR, 964.5–1331), with a similar interval from index infection in the CRH cohort. Complete cohort details can be found in Table 1. All LC participants completed the C19-YRSm. This demonstrated a high level of symptom burden, with significant worsening across all symptom domains when compared with the health state before SARS-CoV-2 infection. Symptom severity score and functional disability were significantly worse in the post–COVID-19 period (Figure 1*A* and 1*B*). Across all symptoms assessed, fatigue showed the largest increase from baseline (Supplementary Figure 2A). The functional impact domain with the largest change was worsening of activities of daily living (household work, leisure/sporting activities; Supplementary Figure 2B). The overall health score, based on a scale from 0 (worst health) to 10 (best health), was significantly worse after COVID-19, with a median 4 as compared with a median 8 prior to infection (Figure 1*C*).

**Table 1.** Characteristics of Patients With Long COVID

	LC Patients (n = 27)	CRH Participants (n = 27)	
Age, y, median (IQR)	52 (17)	46 (22)	*z* = 1.59, *P* = .11
Sex, female, n (%)	19 (70)	18 (67)	χ^2^ = 0.04, *P* = .84
Comorbidity number, median (IQR)	1 (2)	0 (1)	*z* = 1.55, *P* = .13
Comorbidity breakdown			
Respiratory	5	2	
Cardiovascular disease	6	4	
Diabetes mellitus	2	2	
Endocrine	6	4	
Lipid disorder	2	3	
Psychiatric	5	2	
Other	14	8	
Co-medication number, median (IQR)	2 (3)	1 (2)	*z* = 1.81, *P* = .07
Co-medication breakdown			
HRT	2	1	
Supplements	10	8	
Lipid lowering	8	5	
Psychotropic	18	3	
Hypoglycemic agents	5	3	
Antihypertensives	8	6	
Anticoagulants	2	0	
Inhaled therapy	3	3	
Other	11	8	
Duration of index infection/long COVID, d, median (SD)	1130 (367)	1192 (601)	*z* = 0.02, *P* = .99
Severity of index infection, n (%)			*z* = 2,16, *P* = .14
WHO Scale 1	25 (93)	27 (100)	
WHO Scale 2	2 (8)		
Current Long COVID symptom areas, n			
Respiratory	17		
Fatigue	26		
Neurocognitive	24		
Pain	20		
Other	11		
Return to work, yes, n (%)	8 (30)		

Differences assessed using Wilcoxon rank-sum and χ^2^ tests, as appropriate.

Abbreviations: LC, long COVID; CRH, COVID-recovered healthy; WHO Scale 1 ambulatory, no activity limitation; WHO Scale 2, ambulatory, activity limitation; IQR, interquartile range; HRT, hormone-replacement therapy.

### Immune Cell Populations, Mitochondrial Mass, and Mitochondrial Function

**Figure 2.**
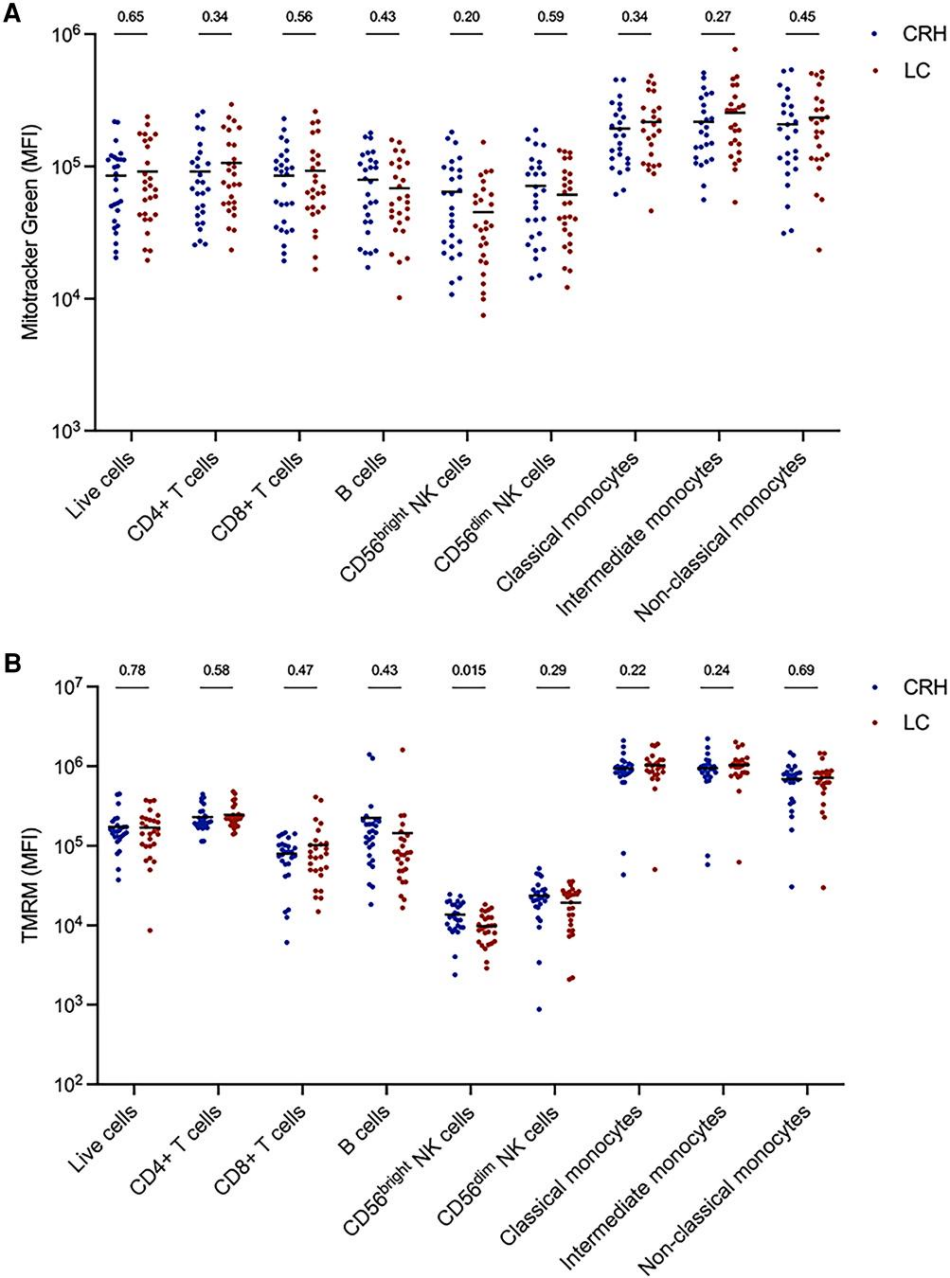

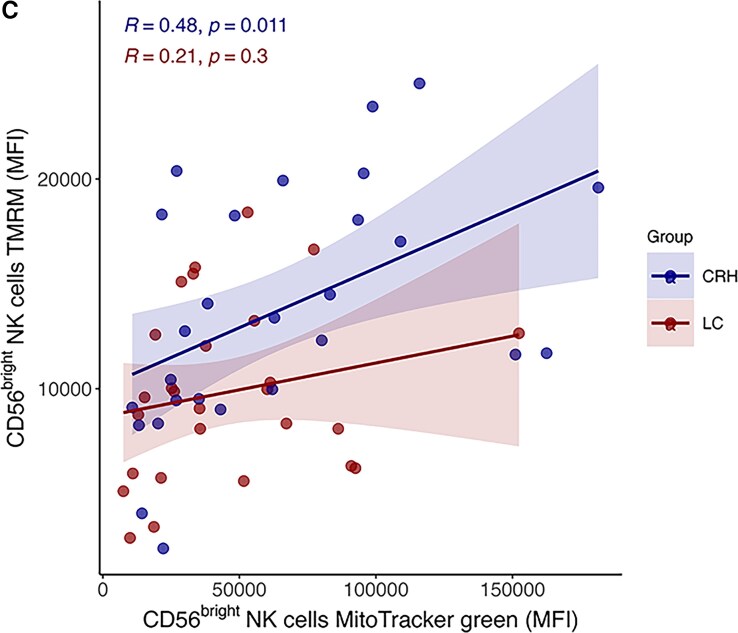
Immune cell mitochondrial assessment. *A* and *B*, Comparison of mitochondrial mass and mitochondrial membrane potential of peripheral blood mononuclear cells in the CRH and LC cohorts. Bars represent the MFI. *C*, Correlation of mitochondrial mass and membrane potential in CD56^bright^ natural killer cells in both groups. Shading represents 95% CI. *P* values were calculated via a generalized linear model adjusted for age and sex. Abbreviations: CRH, COVID-19 recovered healthy; LC, long COVID; MFI, median fluorescent intensity; R, rate ratio; TMRM, tetramethylrhodamine methyl ester.

Cell counts, cell percentages, MMP, and mitochondrial mass of the main circulating immune cell populations in the LC and CRH cohorts were assessed. Immune cell counts (Supplementary Figure 3A) and immune cell percentages (Supplementary Figure 3B) were similar in both groups, as was mitochondrial mass (Figure 2*A*). However, individuals with LC had significantly lower MMP in CD56^bright^ natural killer (NK) cells, suggesting mitochondrial dysfunction (Figure 2*B*). Moreover, the MMP in CD56^bright^ NK cells from patients with LC, in contrast with the CRH cohort, did not correlate with mitochondrial mass, further indicating altered mitochondrial metabolism in these cells (Figure 2*C*).

### Associations Between Symptom Severity and Cytokine Levels Upon Whole Blood Stimulations

**Figure 3.**
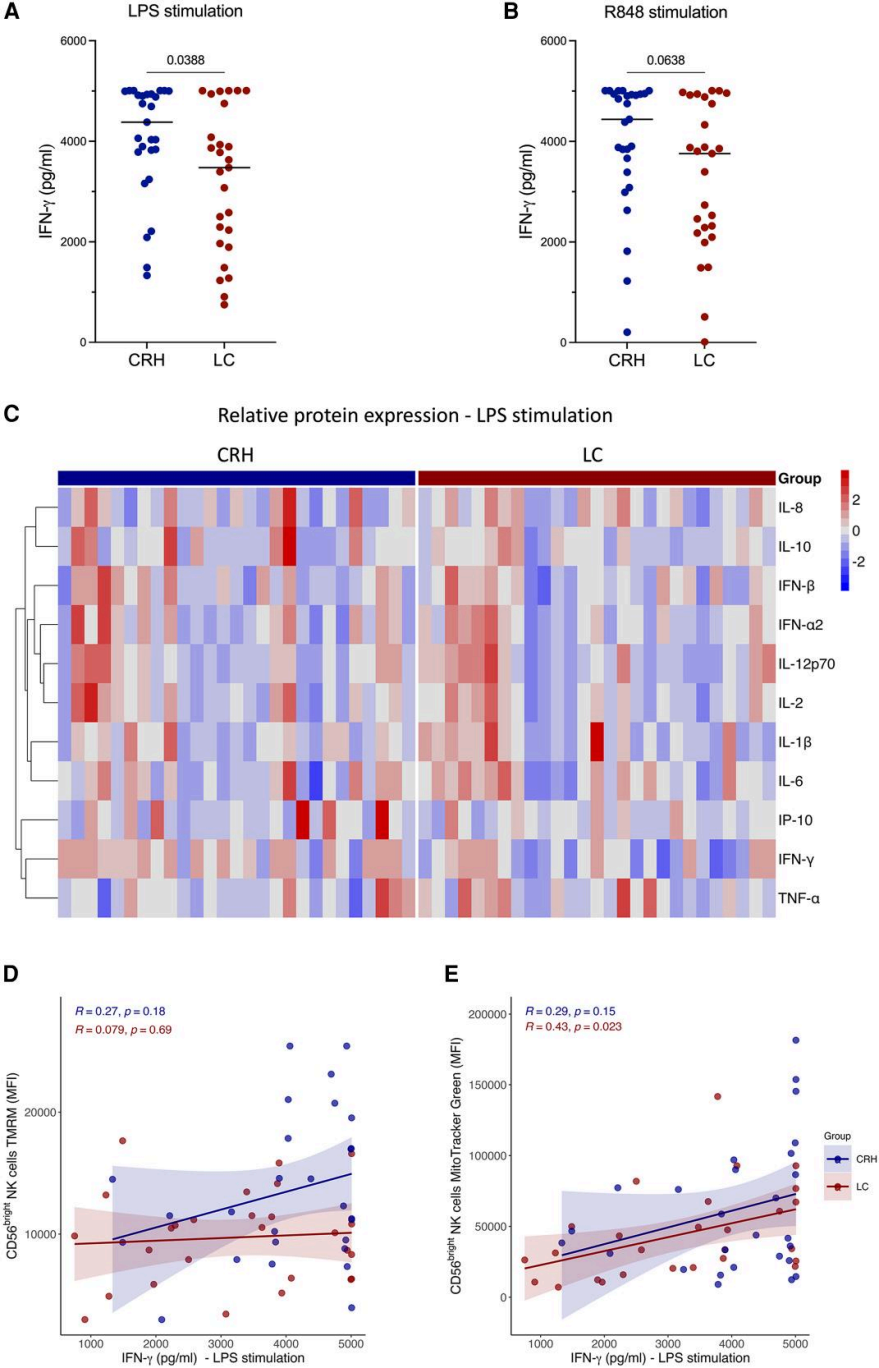
Cytokine responses to LPS and R848. *A* and *B*, IFN-γ protein levels are lower in LC vs CRH cohorts following LPS or R848 whole blood stimulation. *C*, Heat map of the 11 cytokines measured in whole blood upon LPS stimulation in the LC and CRH cohorts. Relative protein expression for each cytokine was calculated by the *z* score normalization: *z* = (*x* − μ)/σ, where *x* is the individual value, μ is the mean, and σ is the SD of each cytokine. *D* and *E*, Correlation of mitochondrial membrane potential and mitochondrial mass of CD56^bright^ natural killer cells and IFN-γ protein levels upon LPS whole blood stimulation. *P* values were calculated via a generalized linear model adjusted for age and sex. Abbreviations: CRH, COVID-19 recovered healthy; LC, long COVID; LPS, lipopolysaccharide; R, rate ratio.

To determine variations in the immune response and potential immune dysregulation in patients with LC, we performed standardized whole blood stimulations with different innate immunostimulants, and cytokine levels were assessed under unstimulated and stimulated conditions [29]. LPS was used to stimulate TLR4, mimicking bacterial infection. Poly I:C, a synthetic analogue of dsRNA, mimics viral infection and activates TLR3. R848 mimics pathogen-associated molecular patterns that activate TLR7 and TLR8. IFN-α2 is a type I interferon and mediates broad adaptive and innate immune responses. IFN-γ production in response to LPS was significantly lower in patients with LC (Figure 3*A*, 3*C*) as compared with the CRH cohort. IFN-γ levels in response to R848 were also lower in patients with LC as compared with the CRH cohort (Figure 3*B*). Other cytokine levels, whether unstimulated or following stimulation with poly I:C or IFN-α2, were similar in both groups. Complete heat maps of cytokine responses following LPS stimulation and other ligands are shown in Figure 3*C* and Supplementary Figure 4. Given the changes seen between the LC and CRH cohorts in CD56^bright^ NK cell mitochondria and IFN-γ responses, we investigated the relationship between IFN-γ and mitochondrial markers in these cells among individuals with LC . There was no association between IFN-γ produced after LPS or R848 stimulation and MMP (Figure 3*D*, Supplementary Figure 5A). However, there was a positive correlation seen with IFN-γ levels and mitochondrial mass (Figure 3*E*, Supplementary Figure 5B).

### Association Among Symptom Scores, Immunometabolic Features, and Cytokine Responses of Circulating Immune Cells in Patients With LC

**Figure 4.**
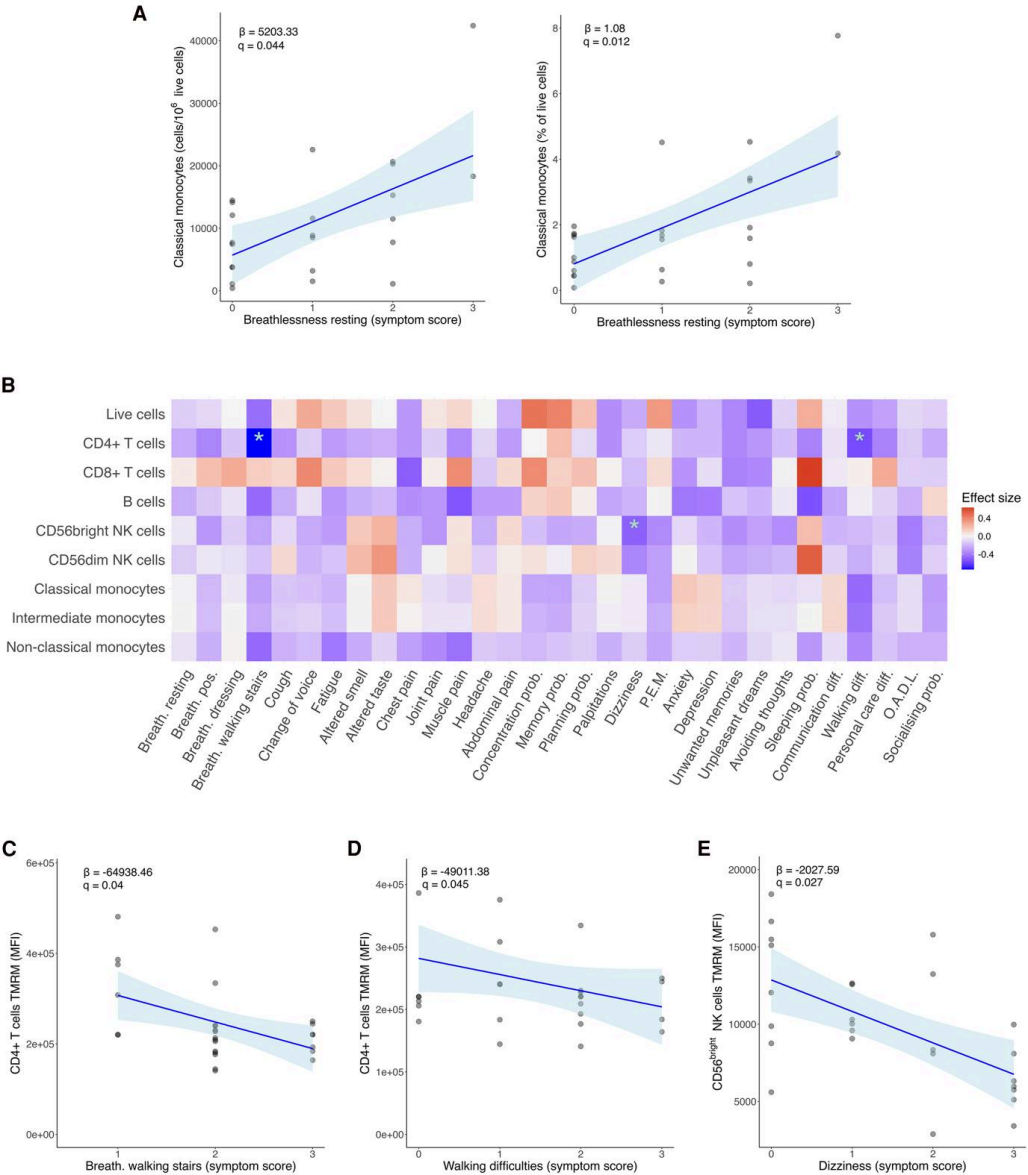
Mitochondrial membrane potential and symptom severity. *A*, Correlation of breathlessness at rest with cell count and cell percentages of classical monocytes. *B*, Heat map of symptoms scores and TMRM MFI. *C*, Correlation of breathlessness–walking stairs symptom score and CD4+ T cells TMRM MFI. *D*, Correlation of walking difficulties symptom score and CD4+ T cells TMRM MFI. *E*, Correlation of dizziness symptom score and CD56^bright^ natural killer cells TMRM MFI. *B*, Effect size was calculated with *z* scores. *A* and *C–E*, Blue line represents the linear model, and the shading shows the 95% CI. β = regression coefficient. Regression coefficients and adjusted *P* values (*q*) were calculated via a generalized linear model adjusted for age and sex. *q* values were corrected for multiple testing via Bonferroni. *q < 0.1. Abbreviations: Breath, breathlessness; Breath pos, breathlessness changing position; diff, difficulties; MFI, median fluorescent intensity; OADL, other activities of daily living; prob, problems; PEM, postexertional malaise; TMRM, tetramethylrhodamine methyl ester.

Given the broad diversity of symptoms associated with LC manifestations, we investigated potential correlations between each symptom burden and the immunometabolic features of circulating immune cells. We found a significant association between the LC symptom of breathlessness in a resting position and the cell counts and percentage of classical monocytes (Figure 4*A*; *q* < 0.1 corrected for multiple testing). There were also significant associations between LC symptoms and cell type–specific MMP (Figure 4*B*). Breathlessness symptoms and difficulties walking were associated with reduced MMP in CD4+ T cells (Figure 4*C* and 4*D*), while worsening dizziness was associated with reduced MMP in CD56^bright^ NK cells (Figure 4*E*; *q* < 0.1 corrected for multiple testing). No significant correlations were observed between symptom burden and the mitochondrial mass of the different circulating immune cells.

**Figure 5.**
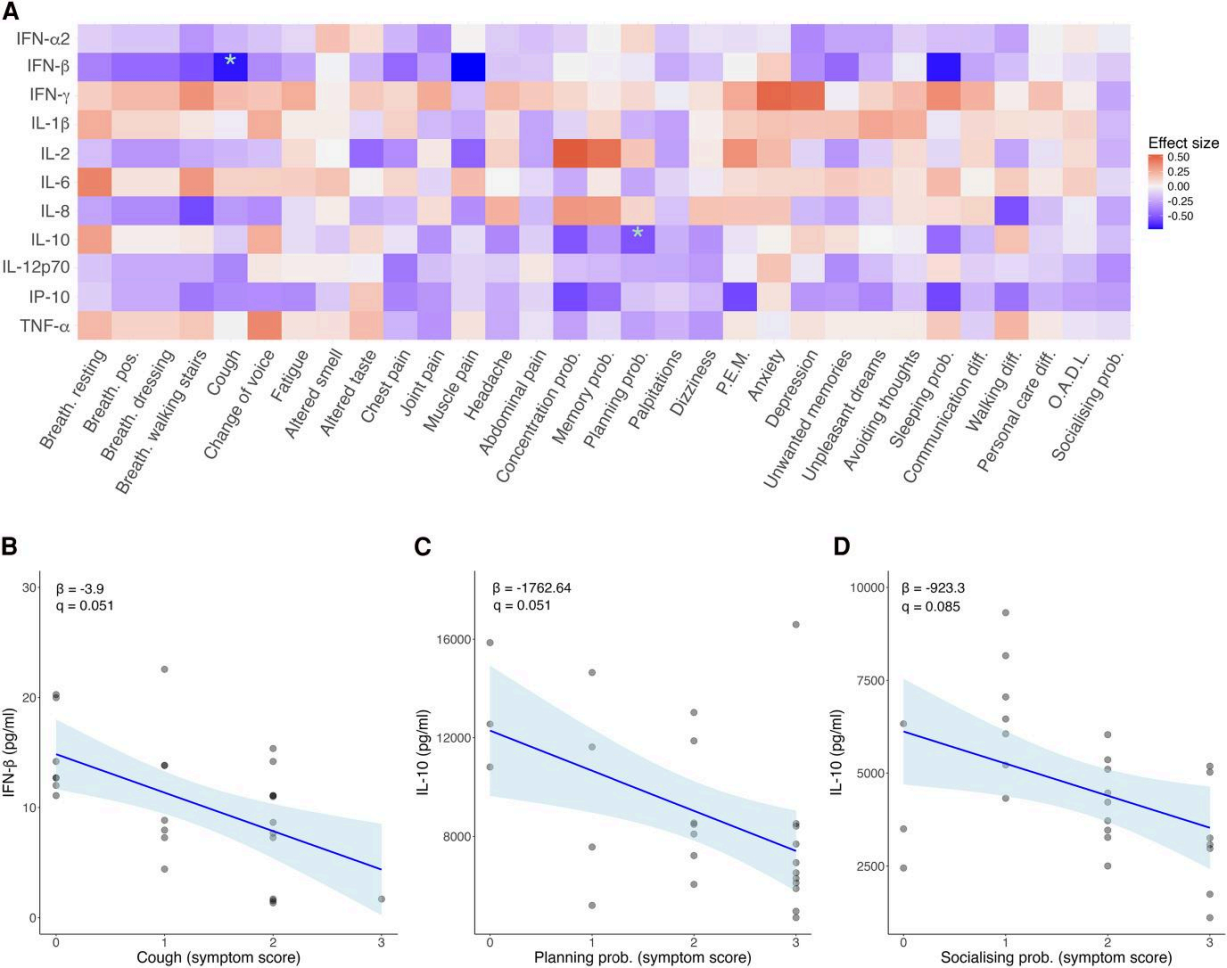
Associations between symptom scores and cytokine responses. *A*, Heat map of symptoms scores and cytokines levels upon R848 stimulation. *B*, Symptom score of cough and IFN-β protein levels upon R848 stimulation. *C*, Symptom score of planning problems and IL-10 upon R848 stimulation. *D*, Symptom score socializing problems and IL-10 protein levels. *A*, Effect size was calculated with *z* scores. *B–D*, The line represents the linear model, and the shading shows the 95% CI. β = regression coefficient. Regression coefficients and adjusted *P* values (*q*) were calculated via a generalized linear model adjusted for age and sex. *q* values were corrected for multiple testing by Bonferroni. *q < 0.1. Abbreviations: Breath, breathlessness; Breath pos, breathlessness changing position; diff, difficulties; OADL, other activities of daily living; prob, problems; PEM, postexertional malaise.

Associations between symptom burden and cytokine levels, unstimulated and stimulated, were investigated. There were no significant associations between cytokine levels and symptom burden under unstimulated conditions or following stimulation with poly I:C or IFN-α2. However, there were significant associations between cytokine levels upon R848 stimulation and symptom scores (Figure 5*A*). Reduced IFN-β responses to R848 were associated with respiratory symptoms, in particular chronic cough (Figure 5*B*). Blunted IL-10 responses to R848 were associated with processing difficulties such as planning (Figure 5*C*), while reduced IL-10 responses following LPS stimulation were associated with worsening difficulties in social interactions (Figure 5*D*).

## DISCUSSION

Here, we provide detailed quantitative clinical phenotyping of individuals with LC using a condition-specific PROM. We demonstrate significant symptom burden in our LC cohort, with LC associated with mitochondrial dysfunctional in CD56^bright^ NK cells and CD4+ lymphocytes, as well as blunted IFN-γ responses to LPS stimulation. Reassuringly, we do not see any significant changes in the proportions or absolute number of immune cell populations between the LC and CRH cohorts. This study highlights the need for a focused clinical assessment to tease the pathogenic LC subtypes apart, as patients may not report the full breadth of symptoms unless asked directly [30, 31]. This detailed clinical data collection and symptom enumeration is essential to evaluating the underlying causes of LC. The inability to distinguish across LC symptom domains may partly explain the limited associations seen with immune correlates in previous studies [32, 33].

The participants in this study have had symptoms consistent with LC for a significant period of time, with the median duration of symptoms being 1118 days (3.1 years). This is important, as previous studies have demonstrated that changes of recovery are highest in the first 6 months after illness onset [34, 35]. The study cohort has established disease, and changes seen are unlikely to represent temporary postinfection changes. Similarly, the cohort is reflective of the overall profile of those with LC, with the majority of working age, predominantly female, and having mild acute disease [36, 37].

We also investigated the relationship between LC symptoms and mitochondrial function in circulating immune cells, as well as cytokine responses to immunostimulants (bacterial and viral pathogen-associated molecular patterns, type I interferon). We identify immune signatures associated with specific LC phenotypes. In particular, increased classical monocyte cell counts and percentages are associated with worsening shortness of breath at rest. Moreover, reduced MMP in CD4+ lymphocytes is associated with worsening shortness of breath and difficulties walking, while reduced CD56^bright^ NK cell MMP is associated with dizziness. Furthermore, blunted IFN-β responses following TLR7/TLR8 and TLR4 stimulation are associated with executive/planning problems and social difficulties, respectively. This builds on the growing knowledge of social stressors on immune responses [38, 39]. Taken collectively, our work supports the concept of LC being an umbrella term for post–COVID-19 symptoms driven by a myriad of pathologic pathways.

Given the role of mitochondrial metabolism and oxidative stress in LC manifestation, we assessed mitochondrial mass and MMP in circulating immune cells [17, 40]. While there were no significant changes in mitochondrial mass, we observed differences in the MMP of CD56^bright^ NK cells from patients with LC as compared with participants who recovered from COVID-19. The decreased MMP may contribute to the dysfunctional cellular activity and impaired cytokine production previously reported for this cell type, through altered cytotoxic responses or reduced pathogen clearance [41–43]. The changes in cytokine production after stimulation but not under unstimulated conditions are indicative of altered immune potential depending on cellular phenotypic features, rather than the percentage of circulating immune cells. While cytokine production in whole blood was measured here, NK cells are primary producers of IFN-γ. Alterations of IFN-γ production by NK cells influence acute COVID-19 severity and viral clearance [44, 45]. Additional studies are needed to definitively identify the immune cell responsible for IFN-γ production in this cohort.

Persistent NK cell impairment after resolution of acute infection may be involved in driving ongoing symptoms. This is supported by our observation that reduced MMP in CD56^bright^ NK cells is associated with symptom syndromes, in particular dizziness. Whether CD56^bright^ NK cells are the main drivers of these symptoms or whether they are the earliest marker of more widespread mitochondrial dysfunction across immune cells remains uncertain. Dysfunction in CD56^bright^ NK cells may indicate loss of immunoregulation. These changes have been seen in other infections, such as hepatitis C, even following successful treatment [46, 47]. The loss of immunoregulatory control may explain the association seen between mitochondrial mass and IFN-γ production after TLR4 and TLR7/TLR8 stimulation. Mitochondrial dysfunction and IFN-γ production have been described in myositis [48]. Future work directly investigating the role played by NK cells will be another important step in our understanding of LC pathogenesis, given the crucial role that NK cells play in controlling chronic viral infections, in particular Epstein-Barr virus and cytomegalovirus [49, 50]. Reactivation of these viruses has been posited to play a role in LC, but definitive evidence is lacking [51, 52].

The other translational findings from our work support the concept of different phenotypes of LC being driven by distinct pathologies [53]. Blunted IL-10 responses to R848 (TLR 7/8 activation) and LPS (TLR4 activation) are associated with worsening executive function and social impairment, respectively. Reduced IFN-β responsiveness to R848 was characteristic of worse cough symptoms. Notably, no associations between symptoms and basal cytokine levels are seen. This reinforces findings from prior studies and suggests that immune cell function upon activation may be the pathologic driver behind some of these symptoms [54].

Mitochondrial changes are poorly described in LC. Previous studies have demonstrated mitochondrial abnormalities in circulating leukocytes following COVID-19 but have not shown associations with symptom burden [55, 56]. Mitochondrial dysfunction has also been associated with an increased likelihood of developing comorbidities after COVID-19 infection [57]. The associations between mitochondrial dysfunction and LC symptom burden identified in this study support the role of mitochondrial metabolism in disease manifestation and suggest targets for therapeutic intervention [58].

Our study is limited in several aspects. We were unable to identify the variant of concern causing infection in each participant and therefore cannot associate disease signatures with specific SARS-CoV-2 subtypes. Cytokines were measured in whole blood, and the specific immune cell responsible for their production was not assessed. Additionally, we did not test for reactivation of chronic viral infections such as Epstein-Barr virus and cytomegalovirus. PROMs are subjective in their nature; participants may have overestimated their premorbid health. This is a single-center study with relatively small numbers of participants in an ethnically homogenous Irish population, which may limit its generalizability. Additionally, while we demonstrate new and important correlates of symptom severity, this study is observational and cannot establish causality. This is an enduring issue in LC studies, with difficulty in establishing disease controls with similar non–COVID-19 secondary issues, including psychosocial stressors. Nonetheless, this study identifies a novel metabolic phenotype of CD56^bright^ NK cells in LC pathology and a specific immune response signature characterized by decreased IFN-γ levels upon activation of TLR4. Further studies are needed to dissect the molecular mechanism behind these alterations, which may unveil therapeutic targets.

## Supplementary Material

ofaf447_Supplementary_Data

## Supplementary Data

Supplementary materials are available at *Open Forum Infectious Diseases* online. Consisting of data provided by the authors to benefit the reader, the posted materials are not copyedited and are the sole responsibility of the authors, so questions or comments should be addressed to the corresponding author.
