# Supplementary material for: Digitally Assessed Long COVID Symptomatology Is Associated With Lymphocyte Mitochondrial Dysfunction and Altered Immune Potential
Source: Open Forum Infect Dis. 2025 Nov 17;12(11):ofaf447. doi: 10.1093/ofid/ofaf447 (PMC12620648; doi:10.1093/ofid/ofaf447)
Supplement: ofaf447_Supplementary_Data [file ofaf447_supplementary_data.zip › supplementary table 1.docx]

**Supplementary Table 1. Antibodies used for circulating immune cells profiling.**

| **Target** | **Fluorochrome** | **Clone** | **Manufacturer** | **Dilution** |
| --- | --- | --- | --- | --- |
| CD3 | BV711 | SK7 | BioLegend | 1:100 |
| CD4 | APC | A162A1 | BioLegend | 1:100 |
| CD8 | BV785 | SK1 | BioLegend | 1:100 |
| CD14 | BV421 | M5E2 | BioLegend | 1:50 |
| CD16 | PE-Cy7 | 3G8 | BD Biosciences | 1:100 |
| CD19 | eFluor 506 | HIB19 | Invitrogen | 1:100 |
| CD56 | BV650 | NCAM16.2 | BD Biosciences | 1:100 |
| HLA-DR | BB700 | G46-6 | BD Biosciences | 1:100 |
| Live/dead | Zombie NIR | N/A | BioLegend | 1:500 |
